# Supplementary material for: Corneal Endothelial-like Cells Derived from Induced Pluripotent Stem Cells for Cell Therapy
Source: Int J Mol Sci. 2023 Aug 4;24(15):12433. doi: 10.3390/ijms241512433 (PMC10418878; doi:10.3390/ijms241512433)
Supplement: Supplementary file 1 [file ijms-24-12433-s001.zip › ijms-2507618-supplementary.pdf]

	Author/ Journal/Year	Sun et al/ Stem Cells Dev/2021	Bosch et al/Front. Bioeng. Biotechnol./2021	Chen et al/ Front Bioeng Biotechnol /2021	Jia et al/ Ann Transl Med/2022
**Differentiation from IPSC to NCC**	**Coating**	Matrigel			Matrigel
	**Media components**	**Basic culture medium:** 80% DMEM/F12 medium, GlutaMAX-I, 20% Knockout™ SR, 1% nonessential amino acids, 0.1 mM 2-mercaptoethanol, 8ng/mL bFGF **NCC induction medium:** Basic culture medium, StemPro neural supplement, 20ng/mL bFGF, 20ng/mL EGF	**NCC induction medium:** DMEM/F12 medium, 1x B-27 supplement, 1x N-2 supplement, 20 ng/mL EGF, 20ng/mL bFGF, 5 ng/mL heparin, 2 mM L-alanyl-L-glutamine	**Induction medium:** DMEM/F12 medium, 1× B-27 supplement, 1x N-2 supplement, 0.1 mM MEM nonessential amino acids, 0.1 mM 2-mercaptoethanol, 10 ng/ml bFGF	**ncEpic hPSC medium:** Commercial xeno-free, feeder-free, chemically defined **NCC cell culture medium:** 50% IMDM, 50% F12 medium, 1× chemically defined lipid concentrate, 5μg/mL transferrin, 5μg/mL FGF2, 5mg/mL BSA, 0.5× B-27 Supplement
	**Small molecules**			2μM SB431542 (TGFβ inhibitor), 2μM DMH1 (BMP receptor inhibitor)	2.5μM non-muscle myosin II inhibitor, 10μM SB431542, 0.5μM CHIR99021 (GSK3 inhibitor)
**Differentiation from NCC to CEC**	**Coating**				Laminin-511
	**Media components**	**CEC induction medium:** Basic culture medium, 8ng/mL bFGF, 0.1x B-27 supplement, 10ng/mL DKK2 (WNT inhibitor), 10ng/mL PDGF-BB	**Human Corneal Endothelial Cell Conditioned Medium:** DMEM high glucose, 5% FBS, 1% GlutaMAX, 2ng/mL bFGF, 0.1mM 2-mercaptoethanol, 10ng/mL heregulin beta, 10ng/mL activin A, 200ng/mL IGF-I, 1% penicillin-streptomycin	**Differentiation medium:** Chemically defined medium, 20 ng/mL EGF	**CEC01 medium:** 50% IMDM, 50% F12 medium, 1× chemically defined lipid concentrate, 5μg/mL transferrin, 5μg/mL insulin, 10ng/mL FGF-1, 5mg/mL BSA, 0.5× B-27 Supplement, 10ng/mL PDGF-BB **CEC02 medium:** 50% IMDM, 50% F12 medium, 1× chemically defined lipid concentrate, 1μg/mL transferrin, 5μg/mL insulin, 5ng/mL FGF-1, 5mg/mL BSA, 0.5× B-27 Supplement, 10ng/mL PDGF-BB
	**Small molecules**	1 mM SB431542, 2.5mM Y27632 (Rock Inhibitor)		A769662 (AMPK activator), AT13148 (Akt inhibitor), 2μM CHIR99021	2.5μM non-muscle myosin II inhibitor, 0.5/1μM XAV939 (WNT inhibitor)
	**Animal model**	Rabbit			
	**Time taken**	26 days	40 days	22 days	24 days

Table S1. Media formulations for differentiation of iPSC to CEC

	**Author/ Journal/Year**	Wagoner et al/Biol. Open/2018	Ali et al/ Investig. Ophthalmol. Vis. Sci./2018	Grönroos et al/Cells/ 2021	Hatou et al/Stem Cell Res/2021
**Differentiation from IPSC to NCC**	**Coating**	Laminin-521	Matrigel	Laminin-521	
	**Media components**	**NCC induction medium:** DMEM/F12 medium, 2% BSA, 2mM GlutaMAX, 0.1mM MEM non-essential amino acid, 1× trace elements A, B and C, 0.1 mM 2-mercaptoethanol, 50µg/mL (+)-sodium L-ascorbate, 10µg/mL transferrin, 10 ng/mL Heregulin β-1, 200ng/mL LONGR3 IGF-I, 8ng/mL FGF2, 0.2% primocin	**mTeSR1 medium:** Basal medium, supplement<br>**Dual Smad inhibitors medium:** 80% DMEM/F12 medium, 20% Knockout™ SR, 1% nonessential amino acids, 0.1mM 2-mercaptoethanol, 1mM L-glutamine, 8ng/mL bFGF	**Serum-free Basal medium:** KnockOut DMEM medium, 15% Knockout™ SR, 2mM GlutaMax-I, 0.1mM 2-mercaptoethanol, 50-U/mL penicillin/streptomycin, 1% nonessential amino acids	**Embryoid body (EB) induction medium:** DMEM/ F12, 1x N-2 supplement, 2% MACS® NeuroBrew-21, 2mM l-alanyl-l-glutamine, 20ng/mL EGF, 1% penicillin/streptomycin<br>**NCC induction medium:** DMEM/ F12, 1x N-2 supplement, 2mM l-alanyl-l-glutamine, 20ng/mL bFGF, 20ng/mL EGF, 5µg/mL heparin, 1% penicillin/streptomycin
	**Small molecules**	10µM SB431542, 3µM CHIR99021	10µM SB431542, 500ng/mL Noggin (BMP inhibitor)	10µM SB431542, 4µM CHIR99021, 10µM retinoic acid	10µM Y27632
**Differentiation from NCC to CEC**	**Coating**			Laminin-521	0.3µg/cm2 iMatrix-511
	**Media components**	**CEnC induction medium:** DMEM/F12 medium, 2% BSA, 2mM GlutaMAX, 0.1 mM MEM non-essential amino acid, 1× trace elements A, B and C, 0.1 mM 2-mercaptoethanol, 50µg/mL (+)-sodium L-ascorbate, 10µg/mL transferrin, 10ng/mL Heregulin β-1, 200ng/mL LONGR3 IGF-I, 8ng/mL FGF2, 0.2% primocin, 0.1× B-27 Supplement, 10ng/mL PDGF-BB, 10ng/ml DKK-2	**Cornea medium:** 80% DMEM-F12, 20% Knockout™ SR, 1% nonessential amino acids, 0.1mM 2-mercaptoethanol, 1mM L-glutamine, 8ng/mL bFGF, 0.1x B-27 supplement, 10ng/mL PDGF-BB, 10ng/mL DKK2	**Serum-free Basal medium:** KnockOut DMEM medium, 15% Knockout™ SR, 2mM GlutaMax-I, 0.1mM 2-mercaptoethanol, 50-U/mL penicillin/streptomycin, 1% nonessential amino acids	**Corneal endothelial differentiation medium (CEDM):** DMEM/F12 medium, 1x Animal origin free ITS supplement, 10ng/mL leukaemia inhibitory factor (IGF1), 10ng/mL insulin-like growth factor 1 (LIF), 2µM Aldosterone/200nM Hydrocortisone
	**Small molecules**			10µM SB431542, 4µM CHIR99021, 5µM (3 days) > 0µM (1 day) retinoic acid	10ng/mL Interleukin 6 (IL6), 5ng/mL Interleukin 11 (IL11), 10ng/mL tumour necrosis factor (TNFα)
	**Animal model**				Monkey
	**Time taken**	~25 to 96 days	20 days	7 days	35 days

Table S2. Media formulations for differentiation of iPSC to CEC
